# Supplementary material for: Comparative multiomics analysis of cell physiological state after culture in a basket bioreactor
Source: Sci Rep. 2022 Nov 23;12:20161. doi: 10.1038/s41598-022-24687-4 (PMC9686226; doi:10.1038/s41598-022-24687-4)
Supplement: Supplementary file 1 — Supplementary Information 1. [file 41598_2022_24687_MOESM1_ESM.zip › raw data/Transcriptomic raw data/6.Enrichment/1.GO/TE1vsTC1/GSEA/TE1/CELLULAR_AMINO_ACID_METABOLIC_PROCESS/CELLULAR_AMINO_ACID_METABOLIC_PROCESS(GO_0006520).html]

Details for gene set CELLULAR\_AMINO\_ACID\_METABOLIC\_PROCESS(GO:0006520)[GSEA]


|  || Dataset | readcount.sample |
| Phenotype | sample.cls |
| Upregulated in class | TE1 |
| GeneSet | CELLULAR\_AMINO\_ACID\_METABOLIC\_PROCESS(GO:0006520) |
| Enrichment Score (ES) | 0.4648465 |
| Normalized Enrichment Score (NES) | 1.8886147 |
| Nominal p-value | 0.0 |
| FDR q-value | 0.18974297 |
| FWER p-Value | 0.494 |
Table: GSEA Results Summary

  

Fig 1: Enrichment plot: CELLULAR\_AMINO\_ACID\_METABOLIC\_PROCESS(GO:0006520)
  
Profile of the Running ES Score & Positions of GeneSet Members on the Rank Ordered List

  

| PROBE | DESCRIPTION   (from dataset) | GENE SYMBOL | GENE\_TITLE | RANK IN GENE LIST | RANK METRIC SCORE | RUNNING ES | CORE ENRICHMENT || 1 | 103219778 | LOC103219778 |  |  | 332 | Infinity | -0.0148 | Yes |
| 2 | 119623429 | LOC119623429 |  |  | 609 | 14.355 | 0.1023 | Yes |
| 3 | 119621285 | LOC119621285 |  |  | 688 | 7.058 | 0.1624 | Yes |
| 4 | 103226506 | ASNS |  |  | 1135 | 3.209 | 0.1713 | Yes |
| 5 | 103219751 | IARS1 |  |  | 1225 | 2.996 | 0.1944 | Yes |
| 6 | 103226274 | GARS1 |  |  | 1248 | 2.924 | 0.2197 | Yes |
| 7 | 103225885 | LOC103225885 |  |  | 1317 | 2.765 | 0.2416 | Yes |
| 8 | 103247264 | SARS1 |  |  | 1470 | 2.514 | 0.2575 | Yes |
| 9 | 103229694 | WARS1 |  |  | 1530 | 2.405 | 0.2765 | Yes |
| 10 | 103232557 | LOC103232557 |  |  | 1545 | 2.400 | 0.2975 | Yes |
| 11 | 103242251 | CARS1 |  |  | 1819 | 2.212 | 0.3052 | Yes |
| 12 | 103238568 | MARS1 |  |  | 1851 | 2.183 | 0.3235 | Yes |
| 13 | 103244220 | NARS1 |  |  | 2207 | 1.982 | 0.3255 | Yes |
| 14 | 103217590 | MARS2 |  |  | 2229 | 1.971 | 0.3423 | Yes |
| 15 | 103244738 | LARS1 |  |  | 2284 | 1.940 | 0.3574 | Yes |
| 16 | 103215103 | TARS1 |  |  | 2297 | 1.936 | 0.3743 | Yes |
| 17 | 103230020 | LOC103230020 |  |  | 2661 | 1.802 | 0.3743 | Yes |
| 18 | 103225174 | YARS1 |  |  | 2735 | 1.782 | 0.3871 | Yes |
| 19 | 103230068 | EPRS1 |  |  | 2879 | 1.739 | 0.3964 | Yes |
| 20 | 103217940 | FARSB |  |  | 3013 | 1.703 | 0.4058 | Yes |
| 21 | 103248920 | NARS2 |  |  | 3083 | 1.690 | 0.4179 | Yes |
| 22 | 103217527 | GLS |  |  | 3103 | 1.685 | 0.4323 | Yes |
| 23 | 103233152 | AARS1 |  |  | 3122 | 1.681 | 0.4466 | Yes |
| 24 | 103248448 | FDXACB1 |  |  | 3535 | 1.602 | 0.4426 | Yes |
| 25 | 103217509 | ASNSD1 |  |  | 3753 | 1.566 | 0.4470 | Yes |
| 26 | 103233321 | KARS1 |  |  | 3934 | 1.536 | 0.4528 | Yes |
| 27 | 103231013 | IARS2 |  |  | 3974 | 1.531 | 0.4648 | Yes |
| 28 | 103244942 | RARS1 |  |  | 4779 | 1.425 | 0.4417 | No |
| 29 | 103220607 | CAD |  |  | 6474 | 1.265 | 0.3772 | No |
| 30 | 103216994 | DARS1 |  |  | 6486 | 1.264 | 0.3881 | No |
| 31 | 103221747 | VARS1 |  |  | 6708 | 1.243 | 0.3895 | No |
| 32 | 103244671 | HARS1 |  |  | 7410 | 1.189 | 0.3688 | No |
| 33 | 103214798 | CARS2 |  |  | 7607 | 1.174 | 0.3706 | No |
| 34 | 103219579 | LOC103219579 |  |  | 7623 | 1.172 | 0.3805 | No |
| 35 | 103230818 | MTR |  |  | 7890 | 1.151 | 0.3789 | No |
| 36 | 103239747 | ASS1 |  |  | 7942 | 1.148 | 0.3870 | No |
| 37 | 103228204 | HGD |  |  | 8404 | 1.126 | 0.3765 | No |
| 38 | 103224110 | WARS2 |  |  | 8852 | 1.094 | 0.3664 | No |
| 39 | 103227615 | QARS1 |  |  | 8953 | 1.089 | 0.3717 | No |
| 40 | 103234649 | SARS2 |  |  | 9349 | 1.063 | 0.3636 | No |
| 41 | 103227605 | DALRD3 |  |  | 9395 | 1.060 | 0.3711 | No |
| 42 | 103236874 | GGH |  |  | 9500 | 1.054 | 0.3760 | No |
| 43 | 103222240 | FARS2 |  |  | 9606 | 1.047 | 0.3807 | No |
| 44 | 103214988 | DTD1 |  |  | 9703 | 1.041 | 0.3858 | No |
| 45 | 103227517 | LARS2 |  |  | 9719 | 1.040 | 0.3945 | No |
| 46 | 103215860 | GLUD1 |  |  | 9739 | 1.039 | 0.4030 | No |
| 47 | 103229732 | EARS2 |  |  | 9816 | 1.034 | 0.4089 | No |
| 48 | 103228400 | LOC103228400 |  |  | 9829 | 1.033 | 0.4177 | No |
| 49 | 103240072 | RARS2 |  |  | 10143 | 1.010 | 0.4128 | No |
| 50 | 103230475 | GLUL |  |  | 10642 | 0.981 | 0.3993 | No |
| 51 | 103244776 | PCYOX1L |  |  | 10790 | 0.970 | 0.4015 | No |
| 52 | 103238531 | GLS2 |  |  | 10844 | 0.967 | 0.4078 | No |
| 53 | 103234002 | FARSA |  |  | 10918 | 0.962 | 0.4132 | No |
| 54 | 103233171 | TAT |  |  | 11137 | 0.948 | 0.4120 | No |
| 55 | 103230563 | DARS2 |  |  | 11422 | 0.931 | 0.4077 | No |
| 56 | 103223875 | DHFR |  |  | 11983 | 0.895 | 0.3907 | No |
| 57 | 103227606 | WDR6 |  |  | 12378 | 0.870 | 0.3809 | No |
| 58 | 103221813 | VARS2 |  |  | 12715 | 0.849 | 0.3735 | No |
| 59 | 103219469 | GLDC |  |  | 13579 | 0.796 | 0.3420 | No |
| 60 | 103224764 | PARS2 |  |  | 13883 | 0.776 | 0.3354 | No |
| 61 | 103215856 | LOC103215856 |  |  | 15122 | 0.700 | 0.2863 | No |
| 62 | 103221433 | AARS2 |  |  | 15706 | 0.662 | 0.2662 | No |
| 63 | 103220134 | LOC103220134 |  |  | 16061 | 0.636 | 0.2561 | No |
| 64 | 103247193 | TARS2 |  |  | 16144 | 0.632 | 0.2581 | No |
| 65 | 103240398 | LOC103240398 |  |  | 16812 | 0.583 | 0.2335 | No |
| 66 | 103219296 | LOC103219296 |  |  | 16979 | 0.571 | 0.2312 | No |
| 67 | 103225598 | MTHFR |  |  | 17224 | 0.557 | 0.2253 | No |
| 68 | 103245223 | FAH |  |  | 18618 | 0.427 | 0.1667 | No |
| 69 | 103220984 | LOC103220984 |  |  | 20294 | 0.226 | 0.0938 | No |
| 70 | 103236430 | TDO2 |  |  | 21000 | 0.059 | 0.0627 | No |
| 71 | 103229772 | ASPG |  |  | 22231 | 0.000 | 0.0077 | No |
Table: GSEA details
[plain text format]

  

Fig 2: CELLULAR\_AMINO\_ACID\_METABOLIC\_PROCESS(GO:0006520)
  
Blue-Pink O' Gram in the Space of the Analyzed GeneSet

  

Fig 3: CELLULAR\_AMINO\_ACID\_METABOLIC\_PROCESS(GO:0006520): Random ES distribution
  
Gene set null distribution of ES for
 **CELLULAR\_AMINO\_ACID\_METABOLIC\_PROCESS(GO:0006520)**
